# Supplementary material for: Deep learning for EEG-based Motor Imagery classification: Accuracy-cost trade-off
Source: PLoS One. 2020 Jun 11;15(6):e0234178. doi: 10.1371/journal.pone.0234178 (PMC7289369; doi:10.1371/journal.pone.0234178)
Supplement: S1 Table — (PDF) [file pone.0234178.s001.pdf]

Dataset 104					
Trial number	Filters	Filter size	Epochs	Learning rate	Test-set Kappa
1	195	3	118	0.000939	0.73787
2	188	5	87	0.001421	0.75458
3	189	3	38	0.000843	0.73803
4	177	3	75	0.000672	0.72963
5	162	3	52	0.001072	0.73796
6	181	3	55	0.000808	0.74652
7	186	3	63	0.000972	0.73782
8	213	3	76	0.00091	0.73782
9	115	3	41	0.001264	0.73803
10	245	3	165	0.001086	0.73782
11	197	3	63	0.000891	0.74652
12	113	3	39	0.001231	0.73827
13	202	3	120	0.001038	0.73791
14	204	5	77	0.000847	0.73762
15	233	3	78	0.000771	0.73791

Table 1: **Hyperparameter description of the CNN models used in the comparisons (dataset 104).**

Dataset 107					
Trial number	Filters	Filter size	Epochs	Learning rate	Test-set Kappa
1	59	9	72	0.001809	0.72255
2	26	11	189	0.002198	0.69704
3	23	11	85	0.001624	0.71398
4	73	3	57	0.001011	0.72229
5	102	3	130	0.001206	0.72224
6	165	3	171	0.001052	0.71393
7	105	11	133	0.001309	0.72240
8	66	13	152	0.002024	0.70552
9	82	5	122	0.002435	0.73917
10	108	5	78	0.000742	0.73907
11	100	5	140	0.00162	0.73066
12	74	9	98	0.00125	0.73071
13	150	3	151	0.001532	0.70552
14	126	11	57	0.00106	0.73066
15	20	15	87	0.001849	0.73066

Table 2: **Hyperparameter description of the CNN models used in the comparisons (dataset 107).**

Dataset 110					
Trial number	Filters	Filter size	Epochs	Learning rate	Test-set Kappa
1	59	17	108	0.000698	0.63753
2	28	11	153	0.002842	0.65442
3	62	9	84	0.001542	0.65442
4	39	17	144	0.001016	0.65435
5	114	7	15	0.000457	0.66297
6	65	13	34	0.000757	0.64606
7	99	15	40	0.000536	0.63763
8	166	13	96	$6.9 \cdot 10^{-5}$	0.65445
9	42	9	135	0.000199	0.65435
10	140	7	162	$5.1 \cdot 10^{-5}$	0.67134
11	159	9	46	0.000328	0.66288
12	44	13	91	0.000687	0.65435
13	75	17	46	0.001307	0.65442
14	81	13	86	0.001391	0.64592
15	170	15	33	0.000859	0.64606

Table 3: **Hyperparameter description of the CNN models used in the comparisons (dataset 110).**
