# Supplementary material for: Deep learning for EEG-based Motor Imagery classification: Accuracy-cost trade-off
Source: PLoS One. 2020 Jun 11;15(6):e0234178. doi: 10.1371/journal.pone.0234178 (PMC7289369; doi:10.1371/journal.pone.0234178)
Supplement: S2 Table — (PDF) [file pone.0234178.s002.pdf]

Dataset 104					
Trial number	Layer 1 units	Layer 2 units	Epochs	Learning rate	Test-set Kappa
1	71	0	88	0.049084	0.68658
2	23	25	78	0.033855	0.72886
3	25	0	95	0.050254	0.67744
4	24	0	82	0.087774	0.67871
5	31	0	60	0.085139	0.67116
6	41	0	62	0.069538	0.68673
7	39	59	45	0.060026	0.69562
8	33	41	93	0.036757	0.70296
9	30	0	92	0.085636	0.69533
10	47	0	43	0.088117	0.67827
11	57	0	69	0.082808	0.70362
12	38	0	60	0.081973	0.67804
13	25	0	94	0.053978	0.68706
14	48	0	70	0.091867	0.68727
15	26	0	95	0.048456	0.70391

Table 1: **Hyperparameter description of the FFNN models used in the comparisons (dataset 104).**

Dataset 107					
Trial number	Layer 1 units	Layer 2 units	Epochs	Learning rate	Test-set Kappa
1	37	99	80	0.028739	0.57963
2	78	0	87	0.108441	0.55483
3	28	0	90	0.064092	0.55491
4	36	0	60	0.190688	0.54711
5	40	0	81	0.155164	0.57106
6	29	46	62	0.070239	0.54651
7	51	78	98	0.035669	0.54651
8	54	42	63	0.048741	0.56356
9	52	41	88	0.117826	0.57915
10	55	0	77	0.133281	0.56298
11	67	59	96	0.042096	0.56323
12	36	59	88	0.094966	0.58881
13	47	90	98	0.054337	0.57138
14	57	36	78	0.119752	0.60403
15	58	92	88	0.061034	0.56248

Table 2: **Hyperparameter description of the FFNN models used in the comparisons (dataset 107).**

Dataset 110					
Trial number	Layer 1 units	Layer 2 units	Epochs	Learning rate	Test-set Kappa
1	28	52	78	0.041539	0.62909
2	79	0	66	0.106168	0.61245
3	38	0	78	0.085864	0.62941
4	95	90	88	0.024561	0.61271
5	30	0	83	0.054989	0.62102
6	52	0	47	0.084462	0.64615
7	39	0	80	0.12204	0.65467
8	40	0	69	0.138589	0.62951
9	28	85	90	0.039441	0.62109
10	54	88	86	0.066	0.64635
11	56	60	80	0.028543	0.63780
12	38	0	83	0.082604	0.62948
13	47	0	95	0.055384	0.64625
14	57	0	67	0.134813	0.62951
15	50	0	88	0.105392	0.63790

Table 3: **Hyperparameter description of the FFNN models used in the comparisons (dataset 110).**
