# Supplementary material for: Deep learning for EEG-based Motor Imagery classification: Accuracy-cost trade-off
Source: PLoS One. 2020 Jun 11;15(6):e0234178. doi: 10.1371/journal.pone.0234178 (PMC7289369; doi:10.1371/journal.pone.0234178)
Supplement: S3 Table — (PDF) [file pone.0234178.s003.pdf]

Dataset 104				
Trial number	Layer units	Epochs	Learning rate	Test-set Kappa
1	42	13	0.010928	0.64506
2	48	15	0.013503	0.65202
3	46	8	0.024349	0.71250
4	46	11	0.019532	0.68638
5	46	15	0.008011	0.66879
6	25	10	0.051127	0.66198
7	20	10	0.047122	0.63593
8	17	9	0.019966	0.66231
9	22	10	0.022322	0.66997
10	20	24	0.005793	0.65343
11	21	6	0.043497	0.69388
12	21	12	0.080816	0.64445
13	24	11	0.018879	0.67761
14	17	8	0.02481	0.70256
15	23	10	0.057033	0.67848

Table 1: **Hyperparameter description of the RNN models used in the comparisons (dataset 104).**

Dataset 107				
Trial number	Layer units	Epochs	Learning rate	Test-set Kappa
1	30	12	0.019647	0.58765
2	38	13	0.006474	0.57971
3	59	14	0.021875	0.55407
4	59	13	0.027082	0.57971
5	59	10	0.023675	0.57963
6	38	9	0.031556	0.55500
7	36	11	0.036092	0.57122
8	42	42	0.037993	0.56339
9	30	12	0.012704	0.56356
10	42	13	0.009591	0.56356
11	52	14	0.01365	0.57187
12	55	17	0.005898	0.57187
13	46	10	0.024401	0.57899
14	54	9	0.029639	0.57995
15	43	15	0.011822	0.56372

Table 2: **Hyperparameter description of the RNN models used in the comparisons (dataset 107).**

Dataset 110				
Trial number	Layer units	Epochs	Learning rate	Test-set Kappa
1	57	19	0.015731	0.61249
2	58	45	0.006507	0.57879
3	59	24	0.011451	0.58745
4	59	10	0.013821	0.61264
5	59	11	0.022024	0.60448
6	13	9	0.089984	0.62084
7	43	10	0.046845	0.61260
8	39	7	0.029582	0.55339
9	35	12	0.031105	0.59576
10	17	19	0.010646	0.57017
11	52	8	0.020652	0.60440
12	55	14	0.007545	0.57073
13	44	9	0.030366	0.59564
14	15	20	0.010497	0.56161
15	42	10	0.015919	0.62944

Table 3: **Hyperparameter description of the RNN models used in the comparisons (dataset 110).**
