# Supplementary material for: Deep learning for EEG-based Motor Imagery classification: Accuracy-cost trade-off
Source: PLoS One. 2020 Jun 11;15(6):e0234178. doi: 10.1371/journal.pone.0234178 (PMC7289369; doi:10.1371/journal.pone.0234178)
Supplement: Attachment — Submitted filename: Review Report.pdf [file pone.0234178.s005.pdf]

The authors have extended their previous work and have used genetic algorithm (GA) in 2 stages to optimize 3 different network models (CNN, FFNN and RNN). The paper lacks novelty, is not clear and has not been evaluated properly. Following are my specific comments:

1. The paper lacks novelty as use of GA for optimization of network parameters is not new. Furthermore, the paper mostly presents the details of the 3 different models, which is already widely available and a detailed information on the actual work is missing from the paper. Hyper-parameter optimization can also be done using Bayesian optimization (for eg. [1]), how does GA compare with Bayesian optimization and what are the advantages of using GA?
2. The authors have used only 3 subject's data to evaluate the performance of their method. It cannot be generalized that the proposed method is good by only evaluating it using data of 3 subjects. More evaluation and analysis is required using larger datasets such as the GigaDB dataset [2].
3. There is no comparison of the proposed method with the existing state-of-the-art-methods. Some of the recent state-of-the-art methods are listed below:
   - S. Li and H. Feng, "EEG Signal Classification Method Based on Feature Priority Analysis and CNN," in *2019 International Conference on Communications, Information System and Computer Engineering (CISCE)*, 2019, pp. 403-406.
   - S. Kumar, A. Sharma, and T. Tsunoda, "Brain wave classification using long short-term memory network based OPTICAL predictor," *Scientific Reports*, vol. 9, p. 9153, 2019.
   - P. Gaur, R. B. Pachori, H. Wang, and G. Prasad, "A multi-class EEG-based BCI classification using multivariate empirical mode decomposition based filtering and Riemannian geometry," *Expert Systems with Applications*, vol. 95, pp. 201-211, 2018.
   - S. Kumar, A. Sharma, and T. Tsunoda, "An improved discriminative filter bank selection approach for motor imagery EEG signal classification using mutual information," *BMC Bioinformatics*, vol. 18, p. 545, December 28 2017.

[1] S. Kumar, A. Sharma, and T. Tsunoda, "Brain wave classification using long short-term memory network based OPTICAL predictor," *Scientific Reports*, vol. 9, p. 9153, 2019.

[2] H. Cho, M. Ahn, S. Ahn, M. Kwon, and S. C. Jun, "EEG datasets for motor imagery brain–computer interface," *GigaScience*, vol. 6, pp. 1-8, 5th April 2017.
