# Supplementary material for: Deep learning for EEG-based Motor Imagery classification: Accuracy-cost trade-off
Source: PLoS One. 2020 Jun 11;15(6):e0234178. doi: 10.1371/journal.pone.0234178 (PMC7289369; doi:10.1371/journal.pone.0234178)
Supplement: Attachment — Submitted filename: ResponseToReviewers.pdf [file pone.0234178.s006.pdf]

Response to the reviewers for the report on PONE-D-19-33145

Summary of Changes

- Reworked the title and abstract to better reflect the contributions of the energy-time analysis
- Added a state-of-the-art review mainly focused on motor imagery with neural networks
- Added a section dedicated to the trade-off between accuracy and model complexity within the same network architecture, since this could be interesting in order to reduce training cost while maintaining similar performance.

Detailed Changes

- **RQ**: Request
- **AA**: Author Answers
- The changes in the original manuscript have been highlighted using the color blue.

Reviewer 1

RQ: In my opinion neither the title of the paper nor abstract properly reflect/represent the use of genetic algorithms or the use automatic tools to the development of neural networks. I think this aspect can be enhanced in both parts, mainly in the abstract.

AA: Thanks to the comments of the reviewer, we have seen that our study benefits from a greater emphasis on the quality-cost analysis. We have reworked the title (*Deep learning for EEG-based Motor Imagery classification: accuracy-cost trade-off*) and the abstract to better reflect the contributions in this aspect.

RQ: The article lacks a literature review about the EGG classification research area because only some papers about mainly belonging to the authors themselves are cited. Authors have to insert this state of art review.

AA: We have included a literature review, mainly concerning motor imagery and neural networks, near the end of the introduction (pages 2-3, lines 54-83).

RQ: Authors do not include a justification and/or an enumeration of the contributions of their paper. I think that using the review of the previous item, authors can fulfill this requirement.

AA: We have added the contributions of our paper just after the literature review (page 3, lines 84-95): *In this paper, we analyze the performance of three types of neural networks (CNN, FFNN, and RNN) after a GA-based hyperparameter optimization procedure. We compare the three architectures among themselves and to previous work in terms of classification accuracy, but we also place emphasis on energy and time consumption during training, which need to be taken into account when creating a BCI framework but are often relegated to the background. Moreover, since the experiments are repeated several times, we take a look at the variability of network sizes within the same architecture to determine the relationship between complexity and accuracy.*

RQ: Authors sentence that use a two-step approach to neural network optimization, however it is not clear how this approach works. Is the best individual obtained from the first stage chosen as the initial individual of the second stage?. This methodology must be clarified as well as the initialization step of the three genetic algorithms used. A figure could enhance these explanations.

AA: A figure (Fig 9) representing the optimization scheme has been added, as well as a brief clarification in the caption: *The parameters from the best individual in the first optimization step are used in the second step*. Regarding the initialization, we have added (page 12, lines 428-430) *The first population is initialized by sampling amounts of active features from a uniform distribution between 1 and 3,600* for feature selection, and (page 12, lines 439-441) *The method used to initialize the first population is also common: the values are sampled from a normal distribution centered at the middle of the allowed ranges* when talking about hyperparameter optimization.

RQ: For FFNN a description for the available activation functions is made but I did not see in the paper the function used in the paper. A justification of this choice should be inserted.

AA: Although the activation functions and some examples are explained in the FFNN section, they can be applied to all networks in the paper. In Tables 4, 6 and 8 the activation function for each type of network is noted, including FFNN, which uses ELU. However, we feel that indeed this can be better clarified by explicitly stating in the text the activation functions used; as such, we have added the following (page 13, lines 450-452): *Finally, each alternative uses the same activation function throughout the experiments: ReLU for CNN and RNN, and ELU for FFNN (we observed that FFNN got stuck in learning with ReLU)*.

RQ: Regarding the parameters of the GA:

- The number of generations for the structure optimization GA and for the learning optimization GA seems a bit low. Have you studied the fitness convergence of these GAs. Please, justify the choice.
- The topology to optimize for CNN is unusual and shallow. CNNs often have more than one layer, at least two, and a pooling layer. Can you justify this choice? Is there another examples of this topology in the literature.
- The only constrain number in the for the FFNN structure is 2 hidden layers. But what is the width of each layer.
- How the constrain structure numbers (250, 2, 60,...) are chosen? A priori, may seems there are not a fair competition.

AA: We think that these are fair questions and, along with answering them here, we have elaborated on some of them in the paper:

- Although the number could be seen as low, the average cross-validation fitness of the population at each generation stabilizes (for CNN, FFNN, and RNN) towards the last generations. We acknowledge that a more in-depth study of the convergence could have yielded more information, but having in mind the time constraints, we focused on making our results more statistically relevant by adding more repetitions.
- The topology for CNN is indeed shallow (an input layer, a convolutional layer, and an output layer). Our early experimentation pointed towards this choice, as the already high training time was substantially increased with additional layers without observed quality improvements. Moreover, aside from the computational constraints, it is interesting to study shallower architectures, as it could be the way to go [1] for neural networks and small datasets due to overfitting.
- The width of the layers in FFNN is not constrained, mostly in order not to interfere with the size ratios between layers that the genetic algorithm finds (in case it decides that two layers are better than one for a particular network). We have extended the previous statement to include a clarification for this (pages 12-13, lines 448-449): *FFNN, on the other hand, is allowed to have up to two hidden layers (of uncapped width to preserve layer size ratios found by the GA).*
- We kept the constraint on training epochs common for the three alternatives (200 epochs), since this hyperparameter is not as network-specific as others (e.g. layer types) and, in our opinion, this makes the comparison a bit fairer. As a consequence, we had to place limitations on the running times by capping the maximum size of the networks with the deadline in mind. This is a good question, and we have also explained it in the paper (page 14, lines 470-474): *The structure constraints of CNN and RNN, which are the two most expensive alternatives, have been set with a balance between freedom for the GA and feasibility in terms of computation times in mind. The limit on the amount of training epochs is the same across the 3 alternatives to make the comparison fairer, as it is a less architecture-specific hyperparameter.*. However, keeping in mind the characteristics of the final models (added to the paper in response to another request), we cannot conclude that these practical limitations have negatively affected the freedom of the GA to search for values. The final values are spread over the allowed intervals with no apparent bias towards more complexity.

RQ: The best individuals (widths, epochs, ...) obtained for each type of NN are not shown. In my opinion it is very interesting showing these best individuals and analyzing the traits of them.

AA: We believe that this is an excellent observation. As such, we have added a new subsection at the end of the experimental results (*4.7 Relationship between model quality and complexity*) that discusses this from the point of view of training cost. It contains three new figures (18, 19, and 20) that depict the sizes of final models for CNN, FFNN, and RNN against their test-set Kappa values, as well as a table (15) with a brief discussion on training epochs. We also reference it in the conclusions (page 21, lines 688-691): *Furthermore, the variability of intra-class cost-quality ratios is also a factor to keep in mind: there are often solutions with very similar accuracies but contrasting training costs, i.e., smaller networks can be competitive too. This could motivate introducing some mechanisms into optimization procedures to try to leverage this insight.*. A breakdown of the final models and the hyperparameter values found by the optimization process can also be seen in the supplementary files **S1 Table** (CNN), **S2 Table** (FFNN), and **S3 Table** (RNN).

Reviewer 2

RQ: The paper lacks novelty as use of GA for optimization of network parameters is not new. Furthermore, the paper mostly presents the details of the 3 different models, which is already widely available and a detailed information on the actual work is missing from the paper. Hyper-parameter optimization can also be done using Bayesian optimization (for eg. [2]), how does GA compare with Bayesian optimization and what are the advantages of using GA?

AA: It is true that we dedicate a sizable portion of our paper to the description of the three main neural network architectures we explore and the genetic algorithm to optimize them. This is because PLOS ONE has a broad thematic scope that reaches readers who may not be acquainted with the concepts. Therefore, in order to make the paper more useful to the reader, we believe that a self-contained introduction is necessary. Regarding Bayesian optimization, we believe that it would be a very interesting approach to evaluate in our datasets, and thus we consider it in our future work (page 21, lines 692-695): *With cost-saving in mind, future work could take a more in-depth look at the differences between networks of the same type but varying sizes, generalize this analysis to different datasets, or tackle the tuning of neural network hyperparameters by using other techniques, such as Bayesian optimization to guide the search in a more informed (and thus, perhaps more efficient) way.*

RQ: The authors have used only 3 subject's data to evaluate the performance of their method. It cannot be generalized that the proposed method is good by only evaluating it using data of 3 subjects. More evaluation and analysis is required using larger datasets such as the GigaDB dataset [3].

AA: The reviewer is certainly right in that more data allows for a better assessment of the problem at hand. However, as this is a requested extension of our original paper (presented at the 2019 IWANN conference), we felt that it was appropriate to keep the experimentation consistent. Furthermore, the fact that there was a deadline in place is also a factor to take into account. Moreover, the subjects from which these three datasets were recorded are particularly good at BCI tasks (which is not always the case), which favors the proper evaluation of methods as it removes a bit of uncertainty originated from noise. We will nonetheless take this suggestion into account for future work.

RQ: There is no comparison of the proposed method with the existing state-of-the-art-methods. Some of the recent state-of-the-art methods are listed below:

- S. Li and H. Feng, "EEG Signal Classification Method Based on Feature Priority Analysis and CNN," in 2019 International Conference on Communications, Information System and Computer Engineering (CISCE), 2019, pp. 403-406.
- S. Kumar, A. Sharma, and T. Tsunoda, "Brain wave classification using long short-term memory network based OPTICAL predictor," Scientific Reports, vol. 9, p. 9153, 2019.
- P. Gaur, R. B. Pachori, H. Wang, and G. Prasad, "A multi-class EEG-based BCI classification using multivariate empirical mode decomposition based filtering and Riemannian geometry,"Expert Systems with Applications, vol. 95, pp. 201-211, 2018.
- S. Kumar, A. Sharma, and T. Tsunoda, "An improved discriminative filter bank selection approach for motor imagery EEG signal classification using mutual information," BMC Bioinformatics, vol. 18, p. 545, December 28 2017.

AA: Unfortunately, our time constraints do not allow us to extend the experimentation to fulfill this requirement. The comparisons would entail either adapting our methods to the datasets used in each of the proposals listed and performing highly time-consuming trials and repetitions, or implementing several methods so that they work with our datasets and performing (potentially) time-consuming trials and repetitions. While we see where the reviewer is coming from, this is not feasible at this moment. However, we appreciate that the reviewer has taken some time to suggest state-of-the-art approaches, and we have included three of them in an improved version of the state-of-the-art (references 24, 25, and 26, pages 2-3, lines 56-63): *The Support Vector Machine (SVM), despite its age, can still produce promising results when paired with the right features: in [24], mutual information is calculated from Common Spatial Pattern (CSP) features to select optimal frequency bands, and dimensionality is further reduced by means of Linear Discriminant Analysis (LDA) before finally classifying the patterns with SVM; in a later study [25], the same authors use LDA for spatial filtering and a Long Short-Term Memory (LSTM) network for temporal filtering before classifying again with SVM. Alternative approaches to classic machine learning also exist, such as classification by Riemannian geometry [26] or by a residual norm-based strategy [27].* In addition, thanks to the comments of the reviewers, we have realized that the energy-time contributions are more relevant than what the original manuscript made them appear, and therefore we have slightly shifted the focus away from the method and towards efficiency matters.

References

[1] F. Lotte, L. Bougrain, A. Cichocki, M. Clerc, M. Congedo, A. Rakotomamonjy, and F. Yger, "A review of classification algorithms for EEG-based brain–computer interfaces: a 10 year

update," *J Neural Eng*, vol. 15, no. 3, 2018.

[2] S. Kumar, A. Sharma, and T. Tsunoda, "Brain wave classification using long short-term memory network based optical predictor," *Scientific Reports*, vol. 9, no. 1, 2019.

[3] H. Cho, M. Ahn, S. Ahn, M. Kwon, and S. Jun, "Eeg datasets for motor imagery brain-computer interface," *GigaScience*, vol. 6, no. 7, pp. 1–8, 2017.
